# Inhibition of Germinal Centre Apoptotic Programmes by Epstein-Barr Virus

**DOI:** 10.1155/2011/829525

**Published:** 2011-10-23

**Authors:** Lindsay C. Spender, Gareth J. Inman

**Affiliations:** Division of Cancer Research, Medical Research Institute, Ninewells Hospital and Medical School, University of Dundee, Dundee DD1 9SY, UK

## Abstract

To establish a persistent latent infection, Epstein-Barr virus (EBV) faces a challenge in that the virus-infected host cell must transit through the germinal centre reaction. This is a site of B cell differentiation where antibody responses are optimised, and the selection criteria for B cells are stringent. The germinal centre environment is harsh, and the vast majority of B cells here die by apoptosis. Only cells receiving adequate survival signals will differentiate fully to be released into the periphery as long-term memory B cells (the site of persistence). In this review, we detail the apoptotic pathways potentially encountered by EBV-infected B cells during the process of infection, and we describe the functions of those EBV-regulated cellular and viral genes that help promote survival of the host B cell.

## 1. Introduction

### 1.1. The Challenge Faced by EBV to Establish a Latent Infection

**Figure 1:**
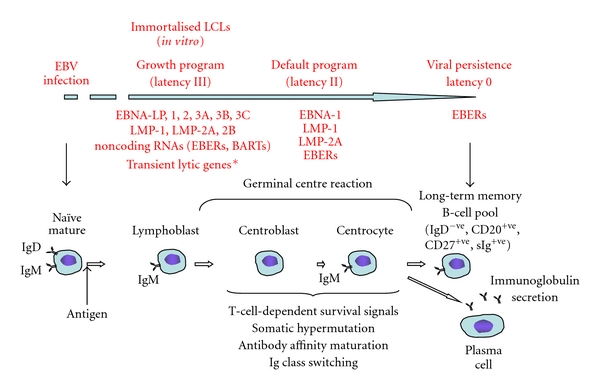
Model of establishment of EBV latency in B cells. EBV infects naïve IgD-positive B cells and drives their proliferation by expression of the viral latency III genes, including the latent membrane proteins LMP1, LMP2A, and LMP2B, the EBV nuclear antigens EBNA1, 2, LP, 3A, 3B, and 3C and noncoding RNA species, the EBV-encoded RNAs (EBERS), and BamHI-A rightward transcripts (BARTs). *In vitro*, these blasts form continuously proliferating immortalised, lymphoblastoid cell lines (LCLs). EBV-infected cells participate in the GC reaction during which time the number of viral gene products expressed decreases due to the downregulation of the viral transcription factor EBNA-2. Following differentiation, long-lived memory B cells emerge as the site of persistent latent infection carrying viral episomal DNA and expressing few viral genes to avoid immune surveillance. *Transient lytic gene expression may occur but without virion production.

Epstein-Barr virus (EBV) persistently infects greater than 90% of the population, and, in the vast majority of cases, the infection remains benign for life. To establish a persistent latent infection, EBV must access the memory B cell compartment and reside within long-lived peripheral B cells [1] where few viral gene products are expressed in order to escape immune detection. One current model suggests that, to establish latency, EBV transmitted in infected saliva first infects IgD+ve naïve B cells within the tonsils of the nasopharyngeal lymphoid system. EBV-infected cells are then thought to express a limited set of viral genes called the latency III or growth program [2] (see Figure 1). More recent evidence, however, has suggested that transient expression of some lytic cycle genes are also required for the early stages of infection but without eliciting virion production (reviewed in [3]). Following infection, an initial phase of naïve B cell activation and proliferation is driven by viral genes which is orchestrated by the viral transcription factor EBNA-2 [4]. EBNA-2 regulates the transcription of all other latent genes as well as a host of cellular genes including the proto-oncogene c-MYC [5, 6]. Inadequate cytotoxic T-cell responses at this stage of infection can lead to infectious mononucleosis (IM) which is characterised by expansion of EBNA-2-positive B cells—a pathological condition not evident in normal carriers. In IM, the normal zonal architecture of the germinal centre (GC) is disrupted due to the extensive proliferation of virally infected cells. In this disease state, there is evidence that EBV may infect and drive the proliferation of cells other than naïve cells (including memory and/or (GC) cells) in order to rapidly spread throughout the B cell population [7]. The extraordinary proliferative capacity of EBV-infected B cells expressing the growth program is evident during *in vitro* culture since infection of resting or GC B cells results in rapid establishment of continuously proliferating immortalised lymphoblastoid cell lines (LCLs) [8]. Through the expression of the latency III genes, EBV infection may also alter the usual phenotypic characteristics of different B cell subsets [9]. However, studies where normal tonsil tissue sections have been dissected and analysed for EBV status and B cell phenotype suggest that, *in vivo*, the expansion of latency III type lymphoblasts is restricted. Instead, the EBV-infected cells enter GCs and actively participate in B cell differentiation. During this time, EBV gene expression gradually becomes more limited due to the downregulation of EBNA-2 (reviewed in [10]). EBV-infected cells expressing only the latency II program (EBNA-1, LMP-1, and LMP-2) have been detected within GCs, and these infected cells retain phenotypic markers of GC centroblasts and centrocytes [11, 12]. These studies suggest that latently infected cells have arisen following differentiation of newly infected blasts which transit normally through the GC reaction before finally emerging into the peripheral memory B cell pool carrying latent episomal viral DNA. Here the virus resides selectively (although not exclusively) within isotyped-switched members of the CD27+ve, IgD−ve memory B cell population [13, 14]. At this stage, the cells are in what is termed the latency 0 stage, expressing only EBV-encoded RNA (EBERs) (Figure 1). It is possible that EBV could access memory B cells via different routes, possibly depending on whether the infection gives rise to the majority of asymptomatic infections or infectious mononucleosis. However, since EBV-infected cells can transit through GCs, it follows that the cells must somehow survive during B cell differentiation in order to establish latency. The GC is a hostile environment for B cells. The greater proportion of them undergoes apoptosis induced as a result of the elimination of all but those B cells expressing the highest affinity immunoglobulins. In the next sections, we discuss the apoptotic signalling pathways that regulate GC B cells and finally consider the virally encoded gene products that may influence host cell survival and thus establishment of viral latency. 

### 1.2. The GC Microenvironment

#### 1.2.1. The GC Reaction

High levels of apoptosis are induced in GCs in order to select effector plasma cells or memory cells capable of expressing high-affinity immunoglobulins of specific subtypes. The process begins with the recognition of antigen by the B cell receptor, along with cognate priming of T-helper cells by antigen presenting dendritic cells. It is generally thought that these activated B cells entering the GC first proliferate rapidly within the “dark zone” (histologically defined as a densely populated area of Ki67^+ve^/CD77^+ve^ centroblasts). Cells accumulate somatic hypermutations in their immunoglobulin genes which has the advantage of potentially increasing the specificity of the BCR for its antigen (affinity maturation), but, also necessitates the elimination of B cells with autoreactive and low-affinity receptors [15]. The centroblasts differentiate further into nonproliferating centrocytes and migrate to the GC “light zone” which contains follicular dendritic cells (FDCs) and T cells. Centrocytes carrying the highest affinity, mutated receptors outcompete others for the limiting amounts of foreign antigen displayed on follicular dendritic cells and for the survival signals provided by helper T cells [16]. In the absence of these survival signals, GC B cells undergo rapid apoptosis. Although the general architecture of the GC is described here, proliferation and apoptosis are not necessarily confined to the dark and light zones, respectively, as cells may traffic between the two regions (reviewed in [17]). In addition, within the light zone, a proportion of centrocytes undergo class switching by DNA recombination to express either IgG, IgA, or IgE and alter the function of their immunoglobulins. In summary, several individual signalling pathways activated during the GC reaction (described in more detail below) form an interconnected web of proapoptotic cues which only the most stringent set of survival criteria can overcome. 

#### 1.2.2. Proapoptotic Signals during T-Cell-Dependent B Cell Responses

**Figure 2:**
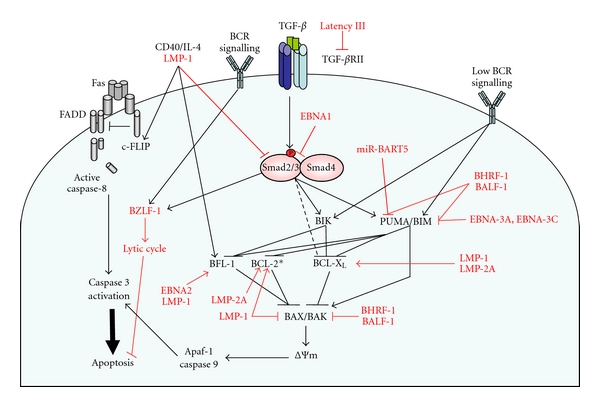
EBV infection impacts on cell death and survival pathways in GC B cells. Interconnected signalling pathways regulate the apoptosis of GC B cells. Proapoptotic signals *via* B cell receptors and the canonical Smad pathway activated by the TGF-*β* receptor control the elimination of unwanted B cells by inducing intrinsic apoptosis. Intrinsic apoptosis is dependent on the activation of the proapoptotic BCL-2 family members BAX and BAK, permeabilisation of the mitochondrial membrane (ΔΨ*m*) and release of proapoptotic factors resulting in the activation of the initiator caspase, caspase 9. An extrinsic apoptotic pathway occurs *via* the death receptor FAS. FAS stimulation results in the formation of the death-inducing signalling complex (DISC) comprised of FAS, the Fas-associated death domain (FADD), and pro-caspase 8. GC B cells, however, have a preformed DISC whose activation is inhibited by binding of the protein cFLIP. The cells are, therefore, dependent on continuous survival signals via CD40 for maintenance of cFLIP levels. CD40 signalling (along with other signals through BLyS (BAFF) and the BCR not shown on this diagram) also induces BCL-2 family members such as BFL-1 and BCL-X_L_ which promote cell survival by inhibiting the intrinsic apoptosis pathway. EBV gene products target multiple points in the apoptosis pathways (shown in red). *In vitro studies of cell lines report the LMP-1-dependent upregulation of BCL-2, however, LMP-1 and BCL-2 expression levels do not correlate in primary tissue.

Apoptotic pathways, responsible for the elimination of B cells within the GC signal through the TGF-*β* receptor, B cell receptors, and FAS (outlined schematically in Figure 2). Using the Burkitt lymphoma model of GC apoptosis, and comparing cells isolated from tonsil tissue, we have shown previously that autocrine TGF-*β* signalling *via* the type 1 TGF-*β* receptor ALK5 contributes to the default apoptotic state of normal GC B cells when they fail to secure survival cues from their microenvironment (death by neglect) [18]. TGF-*β* causes cell death independently of the death receptors FAS and TRAIL [19], by inducing the intrinsic apoptosis pathway. Intrinsic apoptosis requires the activation of two members of the BCL-2 family of apoptosis regulators, BAX, and BAK. These proteins reside in mitochondrial membranes and are responsible for regulating membrane permeability, the release of apoptotic factors into the cytoplasm, and ultimately the activation of an initiator of the caspase cascade (caspase 9). As well as regulating GC B cell homeostasis in the normal GC microenvironment, TGF-*β* signalling is also required for IgA class switching and secretion [20–22]. Proapoptotic signals are also received following the activation of the B cell antigen receptor (BCR) in the absence of T-cell help. This negative selection process is induced as a result of weak or inappropriate BCR ligation and is critical for eliminating B cells carrying autoreactive or low-affinity B cell receptors which can arise due to somatic hypermutation and class switching of immunoglobulin genes. In this context, signalling through the B cell receptor, like TGF-*β*, induces intrinsic apoptosis. An alternative “extrinsic,” FAS-dependent apoptosis pathway also causes spontaneous apoptosis in cells lacking sufficient T-cell help during differentiation. 

Coincidentally ligation of the TGF-*β* and BCR receptors results in the induction of the viral lytic cycle by activating the latent-lytic switch gene BZLF-1 [23, 24]. The BZLF-1 promoter contains multiple Smad-binding elements which act in concert to induce gene transcription [25]. Once established, the lytic programme in its own right protects B cells from apoptosis through late gene expression [26], but the function of BZLF-1 is context dependent. Productive viral infection only occurs after BZLF-1 expression when the viral DNA is methylated (i.e., after latent infection is already established) [27]. 

At what point EBV blocks an apoptotic signalling pathway, whether at the receptor level or downstream at the level of the effector proteins inducing apoptosis, may be determined by the various stages of virus cycle and/or differentiation state of the host cell. The mechanisms of apoptosis induction and the various means employed by EBV to abrogate apoptosis are discussed below.

### 1.3. Mechanisms of Apoptosis Induction

#### 1.3.1. TGF-*β* Signalling Pathway in B Cell Apoptosis

TGF-*β* signals by enabling the formation of a heterotetrameric complex of the high-affinity type II receptor (T*β*RII) and the type I receptor, ALK5. T*β*RII is a constitutively active serine threonine kinase which, upon receptor complex formation, phosphorylates and activates ALK5, inducing a signalling cascade via the canonical Smad pathway and/or several non-Smad pathways [28, 29]. TGF-*β* stimulation of ALK5 results in c-terminal phosphorylation of the receptor-regulated Smads, Smad2 (ser465/476) and Smad3 (ser433/435). Following phosphorylation, Smads 2 and 3 bind to the co-Smad, Smad4, and the resulting heteroligoomeric complexes accumulate within the nucleus to both positively and negatively regulate target gene expression [30]. Phosphorylated (activated) Smad2 has been detected by immunohistochemistry within sections of dark and light zones of GC reactions [18]. In centroblasts isolated from GCs, several of the apoptotic genes regulated by TGF-*β* signalling have been characterised and include members of the BCL-2 family acting upstream of BAX and BAK in the intrinsic apoptosis pathway. 

The BCL-2 family members which tightly regulate the function of BAX and BAK include the prosurvival factors BCL-2, and its homologues, BCL-X_L_, MCL-1, BFL-1, BOO, and BCL-w. Other members of the family, sharing one region of homology with BCL-2 (BH3-only proteins) (BIK, BID, NOXA, BIM, BAD, HRK, PUMA, and BMF), are proapoptotic. Direct “activators” of BAX and BAK [31] include BIM, tBID (the active, truncated form of BID), and PUMA [31–33] which activate BAX/BAK by direct binding in response to apoptotic stimuli. The prosurvival factors like BCL-2 prevent apoptosis by sequestering these “activator” proteins, but they themselves may be inhibited by interaction with specific BH3-only proteins [34]. These proteins are often referred to as apoptosis “sensitisers” which may either free activators from the prosurvival factors to enable BAX/BAK activation [35] or, alternatively, block the direct interaction of prosurvival factors with BAX and BAK [36]. The mechanism of action of TGF-*β* in centroblasts and Burkitt's lymphoma lines involves the induction of an apoptotic program via transcriptional upregulation of the proapoptotic BH3-only proteins PUMA (our unpublished observations) and BIK, while also downregulating the prosurvival factor BCL-X_L_. The increase in BH3-only proteins and the loss of BCL-X_L_ expression lead to mitochondrial membrane depolarisation and intrinsic apoptosis [18]. 

#### 1.3.2. Mechanisms of BCR and FAS-Induced Apoptosis

Like TGF-*β*, signalling via the BCR causes an increase in BH3-only protein expression and induces the intrinsic apoptosis pathway. The proapoptotic proteins BIK and BIM are both induced by BCR signalling. BIM is able to bind all BCL-2 prosurvival proteins making it a highly potent inducer of apoptosis and a critical factor in the homeostatic control of B cells. Its regulation by the BCR is complex involving both transcriptional and posttranslational mechanisms (reviewed in [37]). FAS, on the other hand, activates the “extrinsic” apoptosis pathway independent of the BCL-2 family and the mitochondrial response. Unusually, GC B cells have a preformed FAS death-inducing signalling complex (DISC) which lacks any requirement for ligand binding for its activation. Instead, the preformed DISC is held in an inactive form complexed with cFLIP_L_. In the absence of adequate GC survival signals from cell-cell contact with T cells and follicular dendritic cells, cFLIP is degraded, the initiator pro-caspase 8 within the complex is activated, and the cells undergo rapid spontaneous apoptosis [38, 39]. Targeting mitochondrial apoptosis during EBV infection would have the advantage of potentially inhibiting both TGF-*β* and BCR-induced death, whereas FAS-induced apoptosis (which is independent of the BCL-2 family and mitochondria) would need to be targeted selectively.

### 1.4. Prevention of BCR, FAS, and TGF-*β*-Induced Apoptosis by EBV

The viral latent membrane protein LMP-1, which is induced by EBNA-2 and expressed during the growth programme, mimics constitutively active CD40. LMP-1 regulates NF-*κ*B activity (amongst other signalling pathways) and, in essence, provides the survival signals usually associated with T-cell help. CD40 signalling *via* NF-*κ*B in GC B cells induces the expression of cFLIP and renders the cells FAS resistant [40]. LMP-1, therefore, provides precisely the signals necessary to counteract FAS-induced apoptosis. 

A second mimic of functional B cell receptors, LMP-2A, is expressed by EBV potentially to inhibit negative selection. EBV LMP-2A functions like its own B cell receptor by constitutively associating with Syk and the Src family of tyrosine kinases [41] normally downstream of BCR signalling. Expressed as part of the growth program, LMP-2A is often detected in tumour biopsies of EBV-related malignancies. The effect of encoding its own BCR mimic is that EBV-infected host cells that have lost the capacity to receive normal BCR-derived survival signals (as a result of deleterious or nonsense mutations in the immunoglobulin genes during GC differentiation) can be rescued from apoptosis [42, 43]. *In vitro* infection of CD77-positive (centroblast) GC B cells with EBV can give rise to LCLs with no surface immunoglobulin [44], while LMP2A expression in B cells of BCR-negative mice is sufficient to maintain GC formation in lymphoid tissues of the gut [45]. BCR-negative GC B cells are, therefore, still capable of survival and proliferation by virtue of their positive EBV status. While it is apparent that BCR-negative B cells can survive *in vitro* and in mouse model systems, there is currently no evidence to suggest that outgrowth of BCR-negative virally infected cells actually occurs in humans in the context of a primary infection with the whole virus. Analysis of immunoglobulin genes expressed within cells of the peripheral memory B cell compartment of IM patients failed to detect any cells expressing defective BCR [14]. In addition, LMP-2A is downregulated during the latency 0 phase in the memory B cell compartment and could, therefore, have no further role in promoting their survival. It seems likely then that LMP-2A would have a role in augmenting survival in cells with weak BCR signalling within the GC [46] and potentially during tumourigenesis of cells carrying other genetic abnormalities. LMP-2A can also block normal BCR signalling in LCLs that retain BCR expression [47]; however, in nontransformed mouse models of BCR activation, B cell survival appears more dependent on LMP-2A-induced activation of the NF-*κ*B transcription factor and NF-*κ*B target gene expression than on preventing BCR signalling [48]. 

EBV-infected cells transiting through GCs within tonsils would undoubtedly encounter TGF-*β* signalling via ALK5. Once immortalised, EBV-infected LCLs are refractory to the inhibitory effects of TGF-*β* signalling. This may, at least in part, be mediated by LMP-1. Treatment of LCLs with antisense to LMP-1 modestly sensitises LCLs to the growth inhibitory effects of TGF-*β* [49] although other studies found no evidence that LMP-1 was either necessary or sufficient to block TGF-*β* responses [50]. In epithelial cells, LMP-1-induced activation of NF-*κ*B interferes with TGF-*β*-induced activation of Smad-responsive reporter constructs [51] while in epithelial carcinoma cells and Hodgkin lymphoma cell lines EBNA-1, the viral gene responsible for maintenance and replication of viral episomal DNA, modulates TGF-*β* signalling by reducing Smad2 levels through enhanced protein turnover [52, 53]. Since the TGF-*β* apoptosis programme is multifactorial, it seems unlikely that one viral protein could be sufficient to disrupt the entire apoptotic response. There are, in fact, numerous points of intersection between apoptosis effectors and EBV-induced survival signals as well as substantial cross-talk between the intrinsic apoptosis pathways of TGF-*β* and the BCR. The viral genes that potentially play a role in blocking both TGF-*β* and BCR-induced apoptosis will therefore be discussed together in the next section. 

### 1.5. Viral Products Disrupting the Expression and Function of Proapoptotic Factors

In some lymphomas associated with EBV infection (Burkitt's lymphoma), insensitivity to TGF-*β* can result from a loss of type II receptor expression [50, 54] which correlates with expression of the latency III program although loss of signalling is not mandatory for antagonising the antiproliferative effects of TGF-*β* [55]. This implies that there must be further checks and balances on TGF-*β* target gene expression and/or function to abrogate its effects. 

In EBV-infected epithelial cells, the viral latent membrane protein LMP-1 is involved in blocking TGF-*β*-mediated antiproliferative effects. LMP-1 can prevent TGF-*β*-induced cell cycle arrest by suppressing the TGF-*β*-induced expression of the transcription factor ATF3. Lack of ATF3 induction enables TGF-*β*-mediated expression of Id1 (which would otherwise be inhibited by ATF-3) [56] and the presence of Id1 inhibits TGF-*β*-induced cytostasis [57]. In B cells, TGF-*β*-induced cytostasis can occur if the apoptotic response is blocked. However, ATF3 does not appear to be a TGF-*β* target gene in B cells, even in the absence of EBV infection. Id1 protein is upregulated by TGF-*β*, but, in B cells, growth arrest can proceed uninterrupted in the presence of Id1 [58]. It, therefore, seems unlikely that LMP-1 regulation of Id1 expression may have a role in interfering with TGF-*β*-mediated growth arrest in B cells although this has not been tested during infection of resting B cells. Further studies are needed using primary infected material to determine whether LMP-1 expression in the early phase of infection overrides the TGF-*β* cytostatic response in B cells. 

#### 1.5.1. Blocking BAX/BAK Function

To potentially combat cell death induced by TGF-*β*, EBV employs a variety of prosurvival mechanisms. These include the production of factors which directly counteract the function or induction of the proapoptotic BH3-only proteins (see Figure 2). Several proteins have been implicated in directly blocking the activation of BAX and BAK whose homooligomerization in the mitochondrial membrane [59] is required for apoptosis. *In vitro* (at least when overexpressed) LMP-1 inhibits BAX promoter activity through the induction of NF-*κ*B activity [60]. EBV also expresses two viral BCL-2 homologues. The two v*Bcl-2* genes, *BHRF-1* and *BALF-1*, are maximally expressed just after infection of primary B cells but are not required once latent infection is established in immortalised cell lines. Both genes however, are essential in prevention of spontaneous apoptosis during the earliest stages of infection [61]. Subsequent structural studies have demonstrated that BHRF-1 binds to BAK as well as a subset of BH3-only proteins including the TGF-*β* target gene PUMA, as well as BID and BIM. Interestingly, recombinant BHRF-1 was unable to associate with the “sensitiser” BH3-only proteins BAD, BIK, BMF, HRK, or NOXA [62]. BHRF-1, therefore, appears to selectively target all three direct BAX/BAK “activators” which should prove an effective strategy to prevent BAX/BAK activation. 

#### 1.5.2. EBV-Mediated Induction of Cellular Prosurvival BCL-2 Family Members

Manipulation of cellular gene expression, such as the prosurvival BCL-2 family members BCL-X_L_, BCL-2, MCL-1 and BFL-1, may also contribute to the inhibition of BAX/BAK activation. GC B cells are usually devoid of BCL-2 expression which is transiently down-regulated in GC B cells during the transition from naïve to memory B cell, while BCL-X_L_ is induced by CD40 or BCR ligation. Elevated levels of BCL-2 family members in the GC caused by EBV would be expected to provide a survival advantage to infected cells. BFL-1 has been reported to be induced by overexpression of EBNA-2 or LMP-1, through different response elements within the *BFL-1* promoter. EBNA-2-dependent transcriptional regulation occurs via a CBF-1/RBP-Jk-binding site [63] while LMP-1-induced expression is dependent on NF-*κ*B activation [64]. LMP-1 is also reported to induce both BCL-2 and MCL-1 expression [65, 66], however, more recent studies *in vivo* using human tissue have found no correlation between LMP-1 expression and expression of BCL-2 [12]. The discrepancy here could be due to the difference between *in vitro* overexpression studies compared with the function of LMP-1 *in vivo* in the context of proportionate, and the correct temporal expression of LMP-1.

*In vivo* experiments using transgenic (LMP2A/HEL-Tg) mice in which B cells express LMP-2A and a specific B cell receptor recognising hen egg lysozyme have now provided useful information regarding the potential role of LMP-2A during BCR activation of EBV-infected cells. There is still the potential caveat that LMP-2A is overexpressed and functions in isolation from potential crosstalk with other EBV latent proteins; nevertheless, this analysis has revealed that LMP-2A mimics BCR survival signals by inducing BCL-2 expression. BCL-2 levels were selectively increased in resting, mature B cells via activation of NK-*κ*B [48]. In another mouse model, LMP-2A has also been shown to induce BCL-X_L_ through constitutive activation of the RAS/PI3K/AKT pathway [67]. The activation of PI3K and elevated BCL-X_L_ expression induced by LMP-2A promoted the survival of BCR-negative primary B cells in the periphery (pre-B cell survival signals); however, BCL-X_L_ was not increased by LMP-2A in BCR-positive mature B cells in LMP2A/HEL-Tg mice. There are, therefore, potential differences in the effects of EBV genes depending on the differentiation status of the host cell. Further *in vivo* studies will help determine which members of the BCL-2 family provide essential prosurvival functions and at what stage of EBV infection *in vivo*. 

#### 1.5.3. Blocking BH3-Only Protein Expression and Function

Acting at a level above in the hierarchy of BCL-2 family proteins controlling apoptosis, EBV exerts considerable control over the function or expression of the BH3-only proteins. BIK and PUMA are both direct target genes of TGF-*β* signalling in B cells. PUMA mRNA is also rapidly and significantly upregulated during spontaneous apoptosis of centroblasts following their isolation and *in vitro* culture (our unpublished observations). PUMA, like BIM, interacts with all prosurvival factors while BIK selectively inhibits BCL-X_L_, BFL-1, or BCL-w [34]. The EBV v-BCL2 homologue BHRF-1 directly binds to PUMA (which could potentially interfere with its interaction with other proteins). In addition, EBV encodes its own microRNA (miR-BART5) [68] and induces the cellular microRNA miR-155 [69] which both target *PUMA *transcripts (discussed below). Over-expression of BHRF-1 has been reported to block apoptosis induced by transient transfection of BIK [70], but it does not appear that the inhibition of BIK function is due to any direct interaction between BIK and BHRF-1 [62]. Instead, the effects of BHRF-1 on BIK function are more than likely due to the inactivation of BAK which, unlike BAX, is required for BIK-mediated apoptosis [71]. Interestingly, BIK also mediates host cell suicide in response to protein synthesis shutoff [71], a process commonly observed following viral infection, and induced by the EBV early lytic gene BGLF5 [72]. BIK is also required for IFN-*γ*-induced cell death in human airway epithelial cells [73]. It is possible, therefore, that blocking BIK function may have an important role in evasion of host immune responses. 

As well as inhibiting PUMA and BIK function, EBV infection results in a loss of BIM expression [74, 75]. EBV activation of ERK1/2 kinase, leading to the phosphorylation of BIM and its subsequent degradation by the proteosome, has been proposed as the mechanism for the posttranslational regulation of BIM protein levels [74]; however, other studies suggest that BIM transcription is inhibited through expression of two powerful viral transcriptional repressors EBNA-3A and EBNA-3C [75]. Both viral proteins are essential for immortalisation of primary B cells. EBNA-3A and EBNA-3C can cooperate with activated HRas in cell transformation assays and negatively regulate the activity of the viral protein EBNA-2 by competing for binding of the cellular DNA-binding protein RBP-J*k*/CBF-1 which is needed to tether EBNA-2 to DNA. In biopsies of EBV-positive Burkitt's lymphomas, the *BIM* promoter is methylated at CpG dinucleotides suggesting that epigenetic repression of BIM could have an important role in tumourigenesis [76]. There is support for this hypothesis from studies in E*μ*-*Myc* transgenic mice which carry a deregulated *Myc* transgene under the control of the Ig enhancer region. Deregulation of Myc resembles the chromosomal translocation event associated with the development of Burkitt's lymphoma. In this model, Bim induction, along with the activation of the ARF/p53 pathway, is important for mediating apoptosis caused by the over-expression of Myc. The induction of Puma following Myc-induced p53 activation also performs an important tumour suppressor function in this model which can be overcome, potentially through the induction of Puma binding BCL-2 prosurvival factors downstream of LMP-2A (described above and reviewed in [77]). The loss of a single allele of *Bim* accelerates the development of the lymphomas demonstrating that Bim also acts as a tumour suppressor in B cells undergoing oncogenic stress [78]. The case for EBNA-3A and EBNA-3C having the potential to promote EBV-associated malignancy in the face of deregulated MYC expression is clear. It has not yet been demonstrated that this function of the EBNA-3s is required for viral transformation of primary cells, but, given that one of the major cellular targets of EBNA-2 is c-MYC as described earlier, it is entirely plausible that EBNA-3A and EBNA-3C-mediated repression of BIM is essential during the early stages of infection to prevent MYC-induced apoptosis. In addition, both EBNA-3A and 3C are implicated in the joint repression of p16^INK4a^ [79] and p14^ARF^ [80] required for LCL proliferation. Down-regulation of BIM following late gene expression may also be required during the viral lytic cycle [81]. 

### 1.6. EBV and miRNAs

EBV both regulates the expression of cellular microRNAs as well as encoding 25 of its' own pre-miRNA's located within noncoding regions of the BHRF-1 and BART genes. These ultimately generate four mature BHRF-1 and 40 mature BART miRNAs. microRNAs are small, single stranded noncoding RNAs which block the translation of complementary target mRNA transcripts or, alternatively, result in mRNA degradation. Both of these functions are mediated by an RNA-induced silencing complex [82]. In-depth analysis of the function of EBV-encoded miRNA using recombinant mutant viruses unable to process the longer pre-miRNAs transcripts revealed that, unlike other herpesviruses such as herpes simplex, cytomegalovirus, and Kaposi's sarcoma viruses (which encode microRNAs to help maintain latency), EBV-produced miRNAs have no role in lytic cycle regulation or in maintaining latent infection. Their role primarily seems to involve protecting newly infected primary B cells from spontaneous apoptosis and promoting proliferation during the early phase of infection. Levels of miR-BHRF1-1 and miR-BHRF1-2-3p are four and two-fold higher, respectively, 5 days after-infection compared with expression in established LCLs. Although still expressed in LCL's, they are of lower abundance and appear largely redundant in this context [83]. The cellular targets of the BART miRNAs whose regulation confers a survival advantage on the newly infected cell are difficult to predict and are as yet unidentified. 

A number of BART miRNA target transcripts, however, are known and include LMP-1 and the cellular proapoptotic gene *PUMA*. The majority of studies on BART miRNAs have been carried out in nasopharyngeal carcinoma where BART expression levels are high, so few functional studies have yet been carried out in a B cell background. Targeting LMP-1 with a virally encoded microRNA suggests that the expression levels of LMP-1 are critical and highlights the importance of interpreting over-expression studies with care. 

### 1.7. EBV and p53

Resting primary B cells lack p53 expression and are insensitive to drug-induced DNA damage. Upon infection with EBV, however, p53 levels increase in line with the levels found in B cells stimulated by mitogen. The resultant LCLs are highly sensitive to the activation of p53 by genotoxic agents and undergo apoptosis rather than a cell cycle arrest [84]. Thus, EBV infection *per se* does not block the upstream signals regulating p53 induction and has no effect on the phosphorylation of p53 in response to DNA cross-linking agents. Latent EBV infection does, however, selectively block the ability of p53 to induce p21 in response to DNA-damaging agents inducing cross-links and distortions (e.g., cisplatin) rather than double-strand breaks (e.g., etoposide and *γ*-irradiation). The outcome of inducing DNA adducts is, therefore, apoptosis rather than p21-mediated cytostasis [85]. There is evidence that EBNA-3C can act as a deubiquitinase which leads to stabilisation of the p53-negative regulator MDM2. The levels of p53 in EBNA-3C over-expressing cells are consequently reduced through p53 degradation [86], although in LCLs, this effect may be diminished by binding of EBNA-LP (EBNA-5) to MDM2 which reportedly blocks its ability to target p53 for degradation [87]. The formation of EBNA-LP/MDM2/p53 complexes has been proposed to block p53-mediated transcription (of p21) and provide an explanation as to how rapidly proliferating LCLs tolerate high levels of wild-type p53 without succumbing to p53-induced cell cycle arrest [87]. During the lytic cycle, BZLF-1 mediates p53 degradation independently of MDM2 function thereby blocking the potential for p53-mediated gene transcription during productive viral infection [88]. 

## 2. Conclusions

Establishment of a persistent latent EBV infection requires that the infected host cell transits through the GC where networks of proapoptotic signalling pathways execute a rigorous selection procedure over the differentiating B cells. Few B cells survive this process to differentiate fully. To ensure that the EBV-infected host cell is one of them, EBV has at its disposal an array of prosurvival mechanisms which can potentially override external stimuli promoting cell death (such as TGF-*β* and activation-induced apoptosis) as well as protecting the cell from oncogenic stresses induced during EBV-driven cell proliferation. By blocking cell death pathways, many of the EBV-encoded proteins also inadvertently support the accumulation of genetic mutation and thereby promote tumourigenesis. Sustained expression of the latency III programme as in post-transplant lymphoproliferative disease or the more restricted viral gene expression patterns in Burkitt's lymphoma (EBNA-1 and occasionally LMP-2A) and Hodgkin lymphoma (LMP-1, LMP-2, and EBNA-1) are also likely to make tumours differentially dependent on the presence of EBV [89]. It is important to understand when and where EBV proteins might act to prevent apoptosis and in what particular circumstances, (e.g., LMP-2A behaves differently in different situations, inducing expression of BCL-X_L_ in cells over-expressing Myc [90], but not in normally infected cells). Due to the scarcity of suitable animal models of EBV infection, many of the studies carried out to date have necessarily used established virally infected cell lines or cells over-expressing single viral genes. The results of functional analysis of viral proteins using these systems may at times conflict with the apparent situation *in vivo*. More detailed analysis of newer animal models and primary human tissue may help resolve some of these discrepancies and aid in the identification of new therapeutic targets in EBV-related diseases.
